# Dietary behaviours and weight management: A thematic analysis of pregnant women's perceptions

**DOI:** 10.1111/mcn.13011

**Published:** 2020-04-30

**Authors:** Caragh Flannery, Mavis Nomsa Mtshede, Sheena McHugh, Ann Ebere Anaba, Emma Clifford, Mairead O'Riordan, Louise C. Kenny, Fionnuala M. McAuliffe, Patricia M. Kearney, Karen Matvienko‐Sikar

**Affiliations:** ^1^ Health Behaviour Change Research Group National University of Ireland Galway Ireland; ^2^ School of Public Health University College Cork Cork Ireland; ^3^ Department of Nutrition and Dietetics SIVUH Cork Ireland; ^4^ Department of Obstetrics and Gynaecology University College Cork Cork Ireland; ^5^ Department of Women's and Children's Health, Faculty of Health and Life Sciences University of Liverpool Liverpool UK; ^6^ UCD Perinatal Research Centre, School of Medicine, National Maternity Hospital University College Dublin Dublin Ireland

**Keywords:** diet, obesity, overweight, pregnancy, qualitative research, weight management

## Abstract

Maternal obesity is associated with increased risk of gestational diabetes and other complications. Although antenatal interventions to help prevent these complications are ongoing, an understanding of overweight and obese pregnant women's opinions and attitudes is lacking. Therefore, this study aims to explore these women's experiences and perceptions of dietary behaviours and weight management during pregnancy. Secondary analysis of qualitative data originally collected to examine lifestyle behaviours in pregnant women was conducted. The data were from a purposive sample of overweight and obese pregnant women attending a public antenatal clinic in Cork, Ireland. The data were explored using thematic analysis. Interviews with 30 overweight and obese pregnant women were analysed. Three themes were developed relating to overweight and obese women's dietary behaviours and weight management perceptions including ‘pregnancy's influence on dietary behaviours’, ‘external influences on dietary behaviours’ and ‘perception of and preferences for weight related advice and resources’. Together these themes reveal women's experiences of diet and how pregnancy factors (physiological changes) and external factors (family and friends) can influence dietary behaviours. Furthermore, perceptions of weight management advice and lack thereof were highlighted with women drawing attention to potential resources for future use during pregnancy. This study provides important insights into overweight and obese pregnant women's dietary behaviours and perceptions of weight management. According to these findings, there is a need for clear and unambiguous information about weight management, acceptable weight gain, food safety and how to achieve a balanced diet.

**Key messages**

- The prevalence of overweight and obesity has increased drastically over the years resulting in more women being obese at the onset of pregnancy.
- Understanding women perceptions of diet and weight management is fundamental to inform the development of effective antenatal dietary and weight management interventions.
- Pregnancy factors such as physiological changes and external factors such as family and friends can influence dietary behaviours.
- There is a need for clear and unambiguous information about weight management, acceptable weight gain, food safety and how to achieve a balanced diet.

## 1 INTRODUCTION

Pregnancy is a key stage in a women's life when dietary habits and weight management are of major importance (Lindsay, Heneghan, McNulty, Brennan, & McAuliffe, 2015). Despite this, the prevalence of overweight and obesity has increased drastically over the years resulting in more women being obese at the onset of pregnancy (Goldstein et al., 2017). In Europe, estimates of the prevalence of maternal obesity among women aged 20 to 39 years range from 30% to 37% (Devlieger et al., 2016). In Ireland, 20%–25% of pregnant women have obesity (Fattah et al., 2010; Lynch, Sexton, Hession, & Morrison, 2008). Overweight is defined as body mass index (BMI) ≥25 kg/m^2^, and obesity is defined as a BMI ≥30 kg/m^2^ which is assessed at the first antenatal consultation (Centre for Public Health Excellence at Nice National Collaborating Centre for Primary, 2006). Overweight, obesity and excessive gestational weight gain are associated with complications such as gestational diabetes mellitus, pre‐eclampsia, caesarean section and pre‐term delivery (Catalano & Ehrenberg, 2006; Lynch et al., 2008). Additionally, maternal obesity is associated with risks to the infant including macrosomia, infant and childhood obesity (Catalano & Ehrenberg, 2006).

Nutrition and weight gain during pregnancy have important implications for subsequent maternal and offspring health (Krasovec & Anderson, 1991). Insufficient nutrient intake during pregnancy, such as increased intake of saturated fat or processed foods, overeating and increased frequency of snacking or decreased frequency of lunch eating during or after the pregnancy, can have a negative impact on health outcomes (Brantsæter et al., 2009; Maslova, Halldorsson, Astrup, & Olsen, 2015; von Ruesten et al., 2014; Wu, Bazer, Cudd, Meininger, & Spencer, 2004). Furthermore, maternal diet has been shown to be associated with foetal growth and birth size (Bouwland‐Both et al., 2013; Knudsen, Orozova‐Bekkevold, Mikkelsen, Wolff, & Olsen, 2008).

The increasing rate of maternal obesity has led to national guideline recommendations for the development of interventions to improve pregnancy outcomes (Yaktine & Rasmussen, 2009). This advice stimulated many clinical trials (Dodd et al., 2014; Poston et al., 2015; Sagedal et al., 2017; Szmeja et al., 2014), predominantly of behavioural interventions addressing diet and physical activity.

Systematic reviews of these trials suggest potential for the prevention of gestational diabetes in women with obesity (Rogozińska, Chamillard, Hitman, Khan, & Thangaratinam, 2015). However, most of these trials have been small and underpowered for clinical outcomes such as gestational diabetes and have focused instead on gestational weight gain (Thangaratinam et al., 2012).

Pregnancy has been identified as an ideal time to promote healthy eating and physical activity as women are more likely to experience strong emotional responses to their pregnancy and be motivated to make changes due to the well‐being of the foetus (Phelan, 2010). Additionally, the continuous contact with health care professionals at antenatal visits provides a vital opportunity to interact regarding diet and weight (Beckham, Urrutia, Sahadeo, Corbie‐Smith, & Nicholson, 2015). Despite this opportunity, dietary advice and information on BMI and weight are insufficient for the majority of pregnant women (Markovic & Natoli, 2009).

Although it may be important to target all pregnant women, some studies suggest that interventions can impact women in healthy weight range differently to those who are overweight and obese (Hui et al., 2006; Jeffries, Shub, Walker, Hiscock, & Permezel, 2009; Phelan et al., 2011; Polley, Wing, & Sims, 2002) and that women in higher BMI categories should be considered independently and provided with more intensive interventions (Phelan et al., 2011). Therefore, it is essential to gain a greater understanding of this high risk populations perspectives in order to inform effective antenatal dietary and weight management interventions (Atkinson & McNamara, 2017; Cavanagh & Chadwick, 2005; Furber & McGowan, 2011). The aim of this study is to explore overweight and obese women's experience and perception of dietary behaviours and weight management during pregnancy.

## 2 METHODS

This is a qualitative study using naturalistic inquiry to provide an interpretive description of previously collected qualitative data (Sandelowski, 2000). The aim of the conducted interviews was to collect rich qualitative data on diet, physical activity, weight management, technology use and future interventions. The focus of the original study was to identify barriers and enablers to physical activity using two theoretical frameworks, the theoretical domains framework and the COM‐B model. The focus and insights provided in the current paper are in relation to pregnant women's perceptions of diet, dietary behaviours and weight management. For this secondary analysis of the previously utilised qualitative data set, two additional researchers joined the study team. The data were used and analysed for a different purpose and provides significantly different insights to the original study.

### 2.1 Study design and population

Secondary data analysis of qualitative interviews collected from a sample of overweight and obese pregnant women at risk of gestational diabetes was conducted. Data were originally collected to examine physical activity behaviour in overweight and obese pregnant women, as previously described (Flannery et al., 2018). In brief, medical chart review by midwife or researcher on site, identified pregnant women with a body mass index (≥25 kg/m^2^) recruited during pregnancy from a public antenatal clinic at Cork University Maternity Hospital (CUMH) , Ireland. Eligible participants were approached and informed about the study by researcher (CF) on site at their antenatal appointment.

### 2.2 Interview process

Face‐to‐face interviews were carried out between June and September 2015 in the antenatal clinic on a day and time suitable for the participant. Interviews were conducted by two researchers: a female PhD researcher (health psychology) with experience in qualitative research (CF) and a female student undertaking a master of public health who was new to qualitative research (MNM). Both researchers have a non‐clinical background and have research interests in women's health and maternal health. Prior to the interviews with the pregnant women, both researchers made an effort to build rapport, chatting informally about the research, the interview process and their backgrounds. Both researchers were also sensitive to the fact that they themselves have no lived experience of pregnancy and how this might influence participants' responses. Written informed consent was obtained from all participants. A semi‐structured interview schedule was developed and was used to facilitate the discussion (Flannery et al., 2018.). It consisted of open‐ended questions and prompts about current lifestyle behaviours (physical activity and diet), challenges to engaging in healthy diet and supports available. The interview schedule and process were piloted by interviewing two pregnant researchers. Following this pilot, changes were made to the interview schedule to further explore women's experiences. Pilot interviews were not included in the final sample as the women were not eligible for inclusion in the study. Interviews were recorded and transcribed verbatim. Data on age, nationality, BMI and gestational age were recorded from medical charts where possible.

### 2.3 Data analysis

Secondary analysis was performed using the interview data from the original study (Flannery et al., 2018 ). A thematic analysis was conducted to develop themes relating to weight management and diet for overweight and obese pregnant women (Braun & Clarke, 2006). NVivo software was used to facilitate data analysis. An inductive approach was used, where transcripts were read and re‐read numerous times by the researcher (CF). Transcripts were coded line‐by‐line by researcher (CF) and a subset of transcripts (*n* = 6) were coded by two co‐authors (MNM, KMS). Following open‐coding, categories were developed, discussed, and synthesised to develop broader overarching themes (CF, MNM, KMS ). Discrepancies regarding the codes, categories and themes were resolved through team discussions (CF, MNM, KMS X). Recruitment continued until no new dimensions, nuances or insights were found (Clarke & Braun, 2020). The purpose and goal of this analysis and pragmatic considerations were discussed with the team.

### 2.4 Ethical considerations

This study is reported according to the consolidated criteria for reporting qualitative research (COREQ) statement (Supporting Information S1). Ethical approval was obtained from the University College Cork Clinical Research Ethics Committee of the Cork Teaching Hospitals (ref: ECM 4(y) 06/1/15).

## 3 RESULTS

Of the 30 women who took part in the interviews, over half were Irish (*n* = 16), BMI ranged between 20 and 40 kg/m^2^, and were between 10 and 39 weeks pregnant at time of interview. See Table 1 for all participant characteristics including age, nationality, BMI and gestational age. Interviews ranged from 23 to 50 min in duration.

**TABLE 1:** Participant characteristics

Nationality	
Chinese	2
French	1
Hungarian	1
Lithuanian	1
Irish	16
Nigerian	5
Sudanese	2
Congolese (Democratic Republic of Congo)	1
Ghanaian	1
Age	
20–29	6
30–39	14
40+	1
Unknown^a^	9
Gestation	
First trimester (0 to 13 weeks)	1
Second trimester (14 to 26 weeks)	8
Third trimester (27 to 40 weeks)	20
Not stated	1
BMI (kg/m^2^)^b^	
Overweight 25–29	12
Obese ≥30	12
Unknown^c^	6
Pregnancy	
Singleton	29
Twins	1

Abbreviation: BMI, body mass index.

a Not recorded from medical chart.

b BMI taken from medical chart (calculated at booking visit by midwife).

c Midwife identified women as overweight and obese from chart but did not record BMI.

**FIGURE 1:**
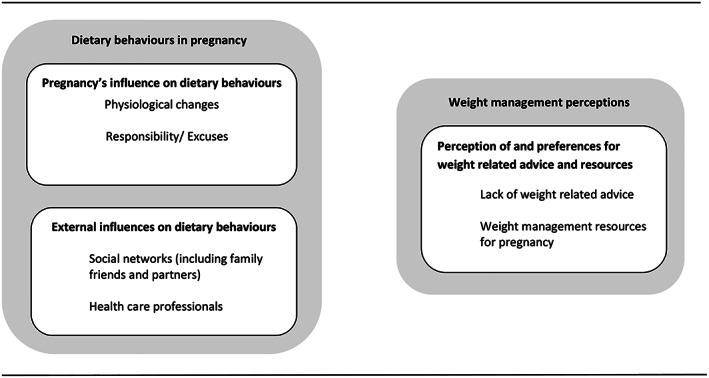
Overweight and obese pregnant woman's dietary behaviours and weight management perceptions

Three major themes were developed that relate to overweight and obese women's dietary behaviours and weight management perceptions during pregnancy: ‘pregnancy's influence on dietary behaviours’, ‘external influences on dietary behaviours’ and ‘perception of and preferences for weight related advice and resources’. Together, the first two themes reflect women's experiences of diet in pregnancy and how pregnancy factors and external factors influence their dietary behaviours. The final theme emphasises overweight and obese women's perceptions of weight management advice and potential resources for future use during pregnancy. The themes are presented in Figure 1.

### 3.1 Pregnancy's influence on dietary behaviours

Pregnancy and pregnancy symptoms influenced women's food preferences and dietary behaviours, with women trying to ‘balance the good with the bad’. For some, pregnancy and its associated ‘physiological changes’ influenced women's food preferences, whereas for others, pregnancy was seen as an ‘excuse’ to indulge. The final subtheme of ‘responsibility’ highlighted that although diet can be influenced by body changes and cravings, the pregnant woman was ultimately responsible for her dietary choices, her health and health of the baby.

### 3.2 Physiological changes

Physiological changes during pregnancy were described by women as a factor that impacted their dietary behaviours. Women who experienced nausea or vomiting discussed how they would restrict certain foods, whereas others experienced food aversions. Women who experienced heartburn and/or indigestion would also limit the consumption of certain foods.

Now I suffer really badly with heart burn … so that would stop me eating a lot of food…Like cereal for example, if I eat Special K I get heart burn. So I am constantly being watchful. (BMI 27 kg/m^2^, age 35)

Furthermore, some women experienced a change in food preferences, an increase in appetite or food cravings. Cravings for specific foods were also common among women in this study with food items such as fast food, crisps and confectionery sweets.

Amm..(pause). Mints, like breath mints. That sort of craving, strange like. Packets of breath mints … I know it's weird. (BMI 30.7 kg/m^2^, age 29)

### 3.3 Responsibility

Some women in this study expressed having a sense of *‘responsibility’* for providing the best for the baby even while experiencing body changes, and food cravings. Furthermore, women discussed *‘making changes’* and being more conscious of their diet and overall health behaviours while pregnant.

I would think way more now about what I am putting into my body. (BMI 30 kg/m^2^, aged 27)

Despite this, for some, pregnancy provided a reason to not make healthy changes ‘… like sure I'm pregnant. I'm going to be big anyway’ (BMI 27 kg/m^2^, aged 35). Women felt that pregnancy could be used as an *‘*excuse’ and was considered a ‘free pass’ in terms of their food choices, with some women ignoring or overlooking healthy eating during pregnancy.

Others described learning from previous pregnancy experiences, where they failed to control their food portions and make healthy food choices. Healthy eating behaviours during pregnancy, especially in terms of the health of their baby were discussed by most women, highlighting that it was reliant on the type of ‘individual’.

I think it definitely depends on the individual, I think it depends on the pregnant mother whether they want to be healthy or not ….(BMI 35.6 kg/m^2^, age 36)

### 3.4 External influences on dietary behaviours

Women's experiences of receiving dietary advice and support throughout pregnancy varied. Women described how their social environment including family, friends and their health care professionals influenced or hindered healthy food choices in pregnancy.

### 3.5 Social influences

Women's friends, family and relatives, in particular the women's partners, played a significant role in providing dietary advice and support, which was often based on their partners own experience or on hearsay. Some women received criticism from their partners regarding their dietary habits and food choices during pregnancy, whereas others felt they were observed and monitored ‘… my husband would be very healthy, he would say why you eating that?’ or ‘My husband watches me every time we go for lunch’. In one case, a woman's husband was concerned about her weight and that she was not eating enough.

… I am not interested in any food and I still have to eat. My husband was giving out to me because my weight was lower, about 5 kg difference compared to when I just got pregnant. (BMI 28.5 kg/m^2^, age unknown)

Partners were not always supportive, with one woman describing her husband as ‘a feeder’ (BMI, 25.6 kg/m^2^, age 35 years). Inputs from other family members were also described; however, advice received was more balanced, allowing for some food indulgences but being watchful of portion size.

My aunt said you are only having a single portion, not the whole thing … I would have had ice‐cream and then a chocolate bar if I was feeling low. Whereas now I know you can't do that, you have to be careful. (BMI 41.7 kg/m^2^, age 28)

For some women, family and friend's encouraged old notions and beliefs which in turn influenced their dietary behaviours ‘… one of my colleagues was with me and whenever we got talking she said, “You're grand you're eating for two, you can have this, you can have that”’ (BMI 27.8 kg/m^2^, age 35).

Despite this, most women did disregard old beliefs ‘… I think people this day and age should know and understand you're not eating for two’. (BMI of 35.6 kg/m^2^, age 36)

### 3.6 Health care professionals

When considering input from health care professionals, women felt that they were not provided with sufficient/adequate information about maintaining a ‘balanced’ diet during pregnancy or diet related health issues. In most cases, diet was referred to quickly by health care professionals at antenatal appointments, emphasising a ‘balanced diet’ and quickly covering the ‘food pyramid’ without any real information or resources to support.

Letting me know about what fruit and veg and about getting the right balance. A balance diet as much as possible, but nothing else. (BMI 35.6 kg/m^2^, age 36)

Diet was usually only discussed in terms of dietary restrictions such as unsafe foods and foods to avoid during pregnancy. Most women felt more emphasis was placed on the clinical aspect of the antenatal visit, ‘it's more the blood pressure, checking the baby and stuff like that’ rather than information and advice on diet.

… my first appointment they said these are the things you do not eat and you are not supposed to have. (BMI 27 kg/m^2^, age 35)

In some cases, women did find dietary advice such as information on food safety and foods to avoid helpful as they were unaware of these essential requirements for pregnancy.

‘she was telling me what to avoid like unpasteurised food and smoked meats and basically all the foods you need to avoid when pregnant, then she was talking about what vitamins I should be taking, like vitamin D and folic acid … just plenty of fluids, obviously avoid caffeine, drinking [alcohol] smoking …’ (BMI 27.6 kg/m^2^, age 32)

### 3.7 Perception of and preferences for weight‐related advice and resources

Despite some receiving information on diet, the majority of women in this study ‘lacked weight related advice’ during pregnancy. Women felt that health care professionals provided limited guidance on appropriate weight gain in pregnancy, with some women enduring uncomfortable and upsetting circumstances when weight was addressed. Furthermore, women highlighted ‘weight management resources for pregnancy’ and how these could be beneficial in supporting a healthy diet and weight management in the future.

### 3.8 Lack of weight‐related advice

Similar to that of diet‐related advice, most women described the weight‐related information in pregnancy as “vague” and “insufficient”. Some women reported trying to get advice from their health care professionals but felt their answers did not support weight or their efforts to manage it. Therefore, women did not feel fully informed or supported in their weight management efforts.

I think the weight should be checked at the same time as they check blood on a more routine basis. Just because every woman is different. (BMI 27.6 kg/m^2^, age 34)

Women who experienced pregnancy‐related health conditions such as gestational diabetes also felt a lack of support in terms of the health problem, their weight and changes in dietary behaviours after diagnosis.

The only reason I knew about diabetes is because I ‘google’ it afterwards…so it's not like your given enough information, there was no leaflet, I definitely wasn't given enough information to just explain what it is [gestational diabetes] or what it could mean for me down the line [in terms of weight and health] …. (BMI 30 kg/m^2^, age 27)

For the few women where weight was addressed, it was brought up during other clinical procedures, and it resulted in a negative and upsetting encounter with their health care professional. In turn, women felt stigmatised and embarrassed and were reluctant for further interactions regarding weight.

I had a very bad experience during my first pregnancy. I was 29 weeks and I went in to see my consultant and asked him if I could find out the sex of the baby but he just pinched my stomach … I felt very upset. I think they turn off when you are a little bit overweight. And they think oh she's after letting herself go …. (BMI 41.7 kg/m^2^, age 28)

### 3.9 Weight management resources for pregnancy

Women mentioned approaches and resources that could potentially support weight management in pregnancy. For example, women acknowledged existing weight loss services such as Weight Watchers, Slimming World and Unislim, which could be tailored for pregnancy to suit their weight and dietary needs.

… if it[diet] could be tailored then to every individual so if you were higher risk, or you weren't. You had certain needs or certain, you know, depending on your own care. (BMI 36 kg/m^2^, age 35)

Others described their experience of ‘tracking calories’ or tracking points for weight watchers. The use of pregnancy and diet smartphone applications as additional support was also discussed as a means of goal setting and self‐monitoring.

If you can link it all together [diet & exercise], how much exercise you should do, what food, how many calories every day and you can track it or like today I did less exercise so tomorrow I have to do more. (BMI 28.5 kg/ m^2^, age not available)

I guess if you had a goal and you had it in your mind that would help you be healthier. (BMI 30 kg/m^2^, age 27)

Furthermore, most women expressed an interest in calorie‐controlled diets or meals plans as a support mechanism that would enable them to make healthy food choices and help to manage weight gain in pregnancy.

If they sit down with you and say here is a meal plan that you can follow and these are the foods you can have. Then you can say to yourself well I can substitute that and this and can add this. It's much easier like that. (BMI 41.7 kg/m^2^ *,* age 28)

## 4 DISCUSSION

This secondary analysis of existing qualitative data emphasises overweight and obese women's perceptions of dietary behaviours and weight management during pregnancy. Three main themes relating to overweight and obese pregnant women's dietary behaviours and weight management perceptions during pregnancy emerged including ‘pregnancy's influence on dietary behaviours’, ‘external influences on dietary behaviours’ and ‘perception of and preferences for weight related advice and resources’. Together, the first two themes reflect women's experiences of diet in pregnancy and how pregnancy factors such as physiological changes and external factors such as family and friends can influence dietary behaviours. The final theme emphasises overweight and obese women's perceptions of weight management advice and lack thereof while also drawing attention to potential supportive resources for future use during pregnancy.

In this population, pregnancy itself had an influence on dietary behaviours. Pregnancy‐related physiological changes such as cravings or food aversions have previously been identified as an influence on dietary behaviour (Quinla & Hill, 2003; Richter, 2003). Although pregnancy‐related symptoms are outside the women's control, they are considered typical features of pregnancy (Quinla & Hill, 2003). Therefore, woman should consider their diets and healthy dietary behaviours preconception and into early pregnancy. Furthermore, as these physiological changes are expected, future interventions need to provide women with the skills to better manage nausea, vomiting, heartburn and indigestion.

Some women revealed ‘responsibility’ as a main reason for being more conscious of their diet and overall health behaviour's while pregnant. In other qualitative studies, this is a commonly reported reason for adopting a healthy diet in pregnancy, the health of the baby and mother (Bianchi et al., 2016). However, for others, pregnancy provided an excuse to indulge. Similar research found that because weight gain can be inevitable during pregnancy (Johnson et al., 2013), some women felt it was a time to relax the rules relating to healthy eating and lifestyle. Previous findings have highlighted perceptions of pregnancy as a time where women could eat large portions and gain weight confidently (Chuang, Velott, & Weisman, 2010; Kraschnewski & Chuang, 2014). For instance, one study involving relatively health‐conscious pregnant women found that pregnancy was viewed as a time when they could take a break from their usual healthy lifestyles (Atkinson, Shaw, & French, 2016). Traditional beliefs such as ‘eating for two’ were still very much evident with women openly sharing their experiences. Changing the culture of “eating for two” in society is necessary to successfully help pregnant women to understand the importance of diet and weight management in pregnancy (Atkinson et al., 2016; Kraschnewski & Chuang, 2014).

Previous research has highlighted that a number of social groups play a significant role in dietary behaviours and weight management during pregnancy; this includes women's mothers (Prichard, Hodder, Hutchinson, & Wilson, 2012), their spouse (Pachucki, Jacques, & Christakis, 2011), as well as friends (Cruwys et al., 2012). Partners, peers and family members have been identified as positive influences on the initiation of healthy eating behaviours (Schaffer & Lia‐Hoagberg, 1997). However, in this study, women's husbands were highlighted in a slightly negative manner in which they criticised or monitored the woman's dietary habits during pregnancy. A systematic review of health behaviour change interventions for couples demonstrated high concordance between partner's health behaviours (Arden‐Close & McGrath, 2017). The review found that interventions for couples led to improvements in cancer screening, increased breastfeeding, reduction in dietary intake, weight loss and increased exercise. Moreover, there is a strong theoretical basis for the effectiveness of couple focused interventions for behaviour change (Arden‐Close & McGrath, 2017). Therefore, the inclusion of husbands/partners in family‐oriented dietary interventions could provide these women with the positive support necessary to promote and encourage healthy eating and weight management in pregnancy.

A lack of reputable dietary information has been reported by other studies, which examined lifestyle behaviours during pregnancy (Gross & Pattison, 2007; Weir et al., 2010). This was echoed in this study as women specified that information was presented on behaviours to avoid, but weight management information was limited. Women felt the information they received lacked focus on favourable foods in pregnancy and weight management. Previous research found that women sought out information themselves due to the lack of information and conflicting advice they received from their midwife on lifestyle behaviours in pregnancy (Brown & Avery, 2012). It is likely that issues of weight are not raised or addressed due its sensitive nature (Blackburn, Stathi, Keogh, & Eccleston, 2015). This lack of advice received by pregnant women is perhaps not surprising given the lack of Irish guidance regarding weight gain in pregnancy (Health Service Executive, 2013). Women also highlighted that they were not regularly weighed at their antenatal appointments. While the National Institute for Health and Care Excellence (NICE) guidelines state that repeated weighing during pregnancy should be confined to circumstances where clinical management is likely to be influenced (guideline, 2007), regular measurement of body weight could be useful for women to highlight weight changes during pregnancy, while also offering health care professionals an opportunity to discuss weight, diet and exercise (Allen‐Walker et al., 2016). Health care professionals have reported that weight monitoring as part of routine antenatal care can reduce women feeling stigmatised (Heslehurst et al., 2013). According to these findings, there is a need for clear and unambiguous information about acceptable weight gain; food safety, how to achieve a balanced diet; the importance of following healthcare professionals' advice and official guidance. This would involve developing and implementing national guidance that could be disseminated by health care professionals. Furthermore, health care professionals will need to be up skilled in order to effectively provide dietary and weight management advice and education to these women.

### 4.1 Strengths and limitations

The thematic approach used to analyse interviews was a strength of this study as it revealed the nuances and experiences of overweight and obese pregnant women in relation to dietary behaviours and weight management. Furthermore, persistent observation was employed between three researchers (two of whom have experience and training in qualitative research). When developing the codes and emerging themes, transcripts were read and reread, codes were revised, checked and verified accordingly and ongoing group discussions were had throughout the analysis. Extensive field notes were documented as an audit trail. Observations about the participants, the setting, researcher's personal diary, coding and theme development were recorded to support credibility and confirmability. All women were recruited through a public antenatal clinic in one maternity hospital setting potentially limiting diversity in study findings; CUMH is a large maternity hospital in the South of Ireland with approximately 8,000 births per year and which services a large representative sample of the Irish population (Cork University Maternity Hospital, 2015 ). Although this ethnically diverse sample of pregnant women shared similar views regarding dietary behaviours and weight management, research is warranted to further assess racial or cultural differences and whether information needs to be tailored for these women.

## 5 CONCLUSION

This study provides important insights into overweight and obese pregnant women's dietary behaviours and perceptions of weight management. Future research is needed to develop strategies that address these perceptions and target specific dietary behaviour's as well as addressing inadequate and conflicting educational messages in pregnancy. Including women's husband/partners in dyadic and family‐oriented interventions could play an important role in promoting and improving diet and weight management for these women. Our results suggest that clear and unambiguous information about acceptable weight gain, food safety and how to achieve a balanced diet is needed. Tailoring existing and effective weight loss programmes for pregnancy might be another potential option to guide woman's dietary habits and help them in their weight management efforts during pregnancy.

## CONFLICTS OF INTEREST

The authors declare that they have no conflicts of interests.

## CONTRIBUTIONS

CF, SMH, PMK, FMA and MB conceived and designed the original study. CF and SMH developed the topic guide and study protocol. MOR and LK facilitated access to the pregnant women. CF and AEA conducted and transcribed the interviews. CF, MNM and KMS guided and developed the aim of this secondary analysis. CF, MNM and KMS coded the transcripts, developed and refined the themes. CF, MNM and KMS wrote the first draft. All authors contributed to successive drafts and the revising of the manuscript. All authors read and approved the final manuscript.

## Supporting information

Supporting Information S1
